# Polarization conversion in cubic Raman crystals

**DOI:** 10.1038/srep41702

**Published:** 2017-02-07

**Authors:** Aaron McKay, Alexander Sabella, Richard P. Mildren

**Affiliations:** 1 MQ Photonics Research Centre, Department of Physics and Astronomy Faculty of Science and Engineering, Macquarie University Sydney New South Wales 2109 Australia; 2 Defence Science and Technology Group, Edinburgh, South Australia 5111, Australia

## Abstract

Nonlinear conversion of unpolarized beams to lower frequencies is generally inefficient in *c*^(2)^ materials, as it is challenging to achieve phase-matching for input ordinary and extraordinary beams simultaneously in the normal dispersion regime. Here, we show that cubic Raman crystals having doubly and triply degenerate (*E* and *F* type) modes provide a method for efficient nonlinear frequency downconversion of an unpolarized beam and yield a linearly polarized output state. Using Mueller calculus, optimal crystal directions for such polarization conversion are determined. Using diamond, an example of an *F*-class Raman crystal, we have verified that such conversion is possible with near quantum-defect-limited slope efficiency and a linear polarization contrast of more than 23.9 dB.

Nonlinear optical frequency conversion by harmonic generation (e.g., second harmonic, sum frequency and difference frequency generation), optical parametric generation and stimulated scattering are fundamental tools for diversifying the emission spectrum of lasers and hence their applications^1^,^2^,^3^. Conversion of unpolarized or partially polarized pumps with high efficiency is an attractive prospect, especially when using high power lasers that may be unpolarized (e.g., many fiber lasers^4^) or those that suffer depolarization due to thermally-induced birefringence at increased power levels^5^,^6^. At the same time, a purely polarized output is crucial to many applications^7^,^8^,^9^, and to enable downstream nonlinear frequency conversion or polarization combination.

In most cases polarized input beams are necessary to achieve nonlinear frequency conversion with high efficiency. One exception is Type II phase matching of second harmonic or sum frequency generation (*o* + *e* → *o*), which converts an unpolarized input to a linearly-polarized output beam at a higher frequency (e.g., green generation in KTP^10^). However, there is no equivalent phase-matching for optical parametric generation of longer wavelengths (frequency downconversion) from an unpolarized input as there is an inherent conflict in satisfying Type I (e.g., *o* → *e* + *e*) and Type II/III (*e* → *o* + o) phase matching conditions simultaneously in the normal dispersion regime. An analogous argument affects unpolarized downconversion by difference frequency generation. Combined second and third order nonlinear processes may offer an approach, however, such methods have only been theoretically proposed and yet to be demonstrated experimentally^11^. If the orthogonal polarization components of the unpolarized input act in a concerted fashion to generate linearly polarized output, conversion efficiencies may be twice that otherwise obtained. Herein, we refer to such cooperative conversion of the orthogonal input components of unpolarized beam^12^ into a pure output polarization mode as “non-trivial” polarization conversion. “Trivial” polarization conversion in this context thus refers to downconversion which converts only a single polarized component to the polarized output with the remaining input polarization components not contributing. Crystals that are orientation patterned for quasi-phase matching provide a method to lift the restrictions imposed by material dispersion. To date, however, there have been no demonstrations of non-trivial downconversion to our knowledge, despite attempts in materials such as orientation-patterned gallium arsenide and as a result downconversion efficiencies remain low^13^,^14^.

We show that stimulated Raman scattering (SRS) in certain cubic crystals simultaneously relaxes the dispersion condition and provides suitable off-axis tensor components for transferring power from orthogonal input polarizations onto a single linear output polarization mode. This allows the optical-to-optical conversion efficiency for unpolarized inputs to be as high as that typically obtained when using linear polarization and potentially as high as the Stokes quantum efficiency. Cubic crystals with two or more degenerate modes are suitable candidates—a category that includes silicon and diamond, which are both extraordinary materials in the context of Raman nonlinear optics^15^,^16^,^17^,^18^,^19^,^20^,^21^,^22^,^23^,^24^,^25^,^26^,^27^. We calculate crystal propagation directions that enable polarization conversion and demonstrate the concept using the example of a Raman laser in diamond.

## Results

### Unpolarised pumping of Raman crystals

The polarization state of an SRS generated beam is determined by the input beam polarization, the symmetries of the Raman medium (which are characterized by the Raman tensors) and its direction through the medium. In the absence of an external polarized seed or strong crystal anisotropy, the Raman gain coefficient (*g*_*R*_) for an output Stokes polarization **e**_*S*_ is given by


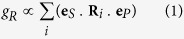


where the input polarization is **e**_*P*_ and **R**_*i*_ are the crystal Raman tensors and the sum is over all degenerate modes^28^. The polarization properties of the initial Stokes mode that develops from spontaneous scattering and the competition between them are dictated by this product sum. For a well-defined input polarization this calculation is straightforward for a given propagation direction and set of pump and Stokes polarization vectors in a Raman medium. However, for partially polarized or unpolarized light a phenomenological approach is needed in which Raman scattering is treated as an ensemble of spontaneous scattering events. Mueller analysis offers one such approach^29^.

Following Chandrasekharan^30^,^31^, the combined effect of the Raman tensors can be expressed by a 4 × 4 matrix, **M**, for a given crystal orientation and scattering direction. The output Stokes vector **S** (note, here the term Stokes is used in the context of polarization states) describing the output polarization state is


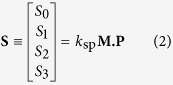


where *k*_sp_ is an appropriate scaling factor for the crystal’s polarisability, and **P** is the pump polarization vector (in Stokes Mueller formalism). The total scatter is proportional to *S*_0_ and the polarized component is 
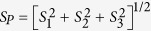
. For a given propagation direction and polarization, the Raman gain is given by ref. 25


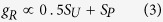


where the unpolarized component of scatter *S*_*U*_ = *S*_0_ − *S*_*P*_. The Raman interactions in cubic crystals can be grouped by their symmetry and degeneracy into three Raman modes classes: *A, E* or *F* describing non-degenerate or totally symmetric vibrations, doubly degenerate with two axes of 3-fold symmetry, and triply degenerate vibration modes with three 2-fold axes, respectively. The output Stokes vectors **S**_*j*_ for Raman scattering from an unpolarized pump beam in the direction collinear with the pump are


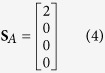



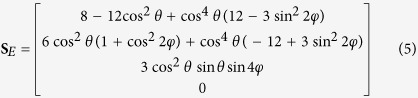



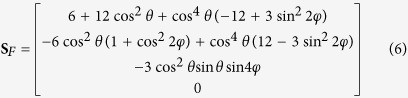


for *j* = *A, E* and *F* Raman modes respectively^31^. The *θ* and *φ* in (5, 6) represent Euler rotations from a <100> oriented cubic crystal about the *z*-axis and then subsequently about the rotated *x*-axis (i.e., the *x*′-axis), respectively^32^. The propagation direction of the pump and Stokes beams is parallel to the initial *y*-axis, and as a consequence the polarization axes are described from the reference *x*-*z* frame and for simplicity termed horizontally (0°) and vertically (±90°) polarized.

### Polarization dependence of spontaneous scattering and Raman gain

**Figure 1:**
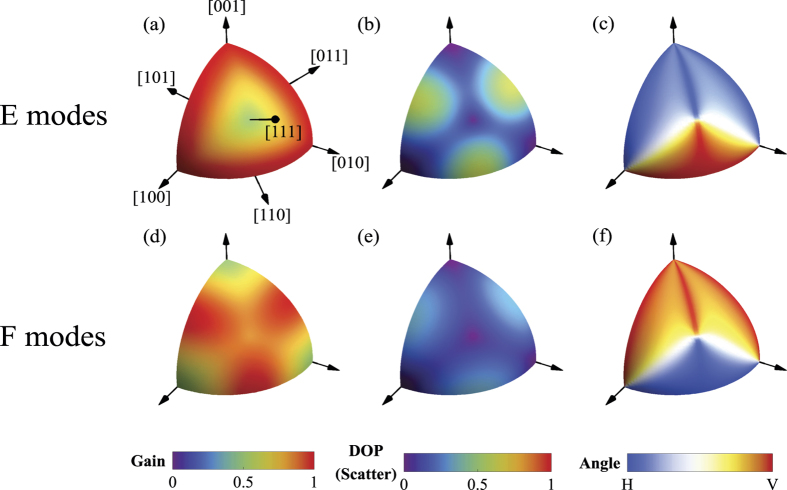
Raman gain (**a,d**), degree of polarization (**b,e**) and direction of the dominant polarization scatter (**c,f**) for *E* (top row: **a,b,c**) and *F* modes (bottom row: **d,e,f**) for unpolarized pumping. Directions of high symmetry are indicated in (**a**). The gain values in (**a,d**) are calculated for the polarization angles identified in (**c,f**) and have been normalized to the Raman gain for horizontally-polarized pumping (i.e., polarized parallel to <100>) along the <110> crystal direction.

The analysis so far enables the gain and the output polarization to be determined for an unpolarized pump beam. Maps for Raman gain calculated from (3), degree of polarization (DOP = *S*_*P*_/(*S*_*P*_ + *S*_*U*_)) and principal direction of the polarized output (Φ = 0.5 arg(*S*_1_ + *iS*_2_)) are shown in Fig. 1 for *E* and *F* Raman modes. The results for *A* Raman modes are not shown as its simple symmetry (i.e, lack of off-axis tensor components) leads to a simple result. For any propagation direction, the gain is uniform for any input polarization^33^ and the output polarization predicted to have a DOP = 1 with polarization in the same direction of the pump in the case of a linearly polarized pump and a DOP = 0 for an unpolarized pump. As a result, *A* Raman modes provide no mechanism for non-trivial polarization conversion.

The output mode of a Raman laser is seeded by the spontaneous scattering and thus has a polarization dictated by the dominant scattered polarization direction shown in Fig. 1(c,f), which has been calculated for non-zero DOP values (Fig. 1(b,e)). Once a Raman laser or SRS threshold condition is attained, gain saturation by pump depletion will ensure that the output will be linearly polarized at this calculated angle. For zero DOP values, the output polarization is predicted to be polarized in a random direction. The threshold (and efficiency) dependence on propagation direction are determined by the calculated Raman gain (3) values shown in Fig. 1(a,d).

For both *E* and *F* modes, the Raman gain maximizes for unpolarized pumping along the <110> directions with values as high as those for purely linear polarized pumping. These directions also provide non-zero DOP and, therefore, produce well-defined linear output polarizations. It is noted that for *F* modes, all directions that have maximum gain coincide with a maximum in the DOP. In contrast, *E* modes have high gain along <100> directions but output with zero DOP. It interesting to note also that, the output polarizations for *E* and *F* modes are orthogonal for a given propagation direction.

### Coupling of input polarizations to the Stokes polarization mode

**Figure 2:**
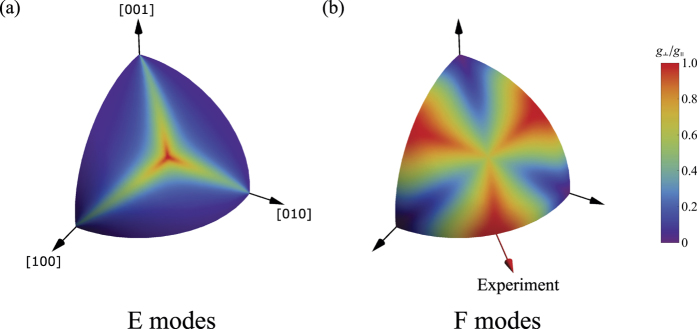
Calculated ratio of gain coefficients (g_⊥_/g_||_) for (**a**) *E* and (**b**) *F* class Raman modes as a function of propagation direction through the crystal. The orientation of diamond used in the experiments is indicated in (**b**).

Although Fig. 1 enables prediction of the output polarization characteristics, for non-trivial polarization conversion there is also a requirement for all input polarizations to be coupled to the output polarization mode. The coupling of the orthogonally polarized input components to the output polarization mode is shown in Fig. 2 for *E* and *F* Raman modes. Here, the ratio of scattering from each orthogonal input state is given by


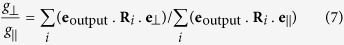


for the output polarization obtained from Fig. 1(c,f). We set the “parallel” component of the unpolarized beam as the pump polarization which gives the highest gain for the Stokes polarization determined from the Mueller analysis for a given crystal orientation. A zero value for (7) indicates there exists one component of the unpolarized pump that is not coupled to the output polarization mode. Hence this polarization component does not contribute to the output, only a maximum of half of the input pump power is converted to the output mode and the SRS threshold is at least twice that of an optimized linearly polarized pump beam. Figure 2 shows that, for *E* modes, equal coupling of both input polarizations is obtained for propagation along a <111> axis. For *F* modes, equal coupling is obtained for propagation directions along and nearby a <110> axis.

### Experiment

**Figure 3:**
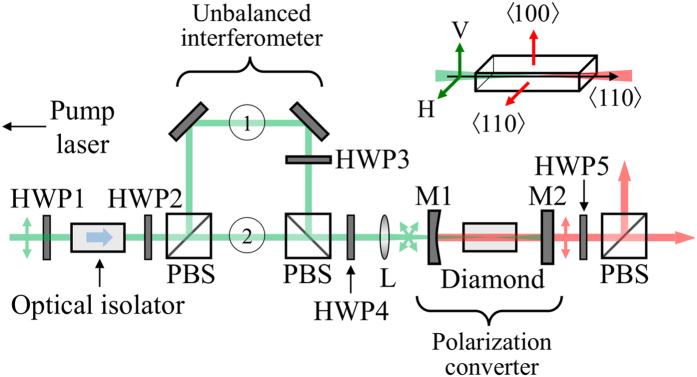
Experiment schematic showing pump (green) and Stokes (red) beam paths. HWP1–5 are half waveplates; PBS are polarizing beam splitters; L is a focusing lens; and, M1 and M2 are resonator mirrors. The inset shows the orientation of the diamond with respect to the beam propagation and horizontal and vertical polarization directions.

Diamond has *F*-class symmetry^34^ and has recently been demonstrated to be a highly promising Raman laser material in terms of efficiency^22^,^35^,^36^,^37^, as well as power^23^,^26^, and wavelength range^35^,^38^,^39^,^40^, and in bulk and waveguide form^27^,^41^. It is thus an excellent candidate for demonstrating efficient polarization conversion. To date, the polarization properties of diamond have only been investigated using purely polarized pump states^22^,^24^,^42^. Here we investigate polarization conversion of an unpolarized nanosecond-pulsed 1-micron beam in <110>-cut diamond. The experimental setup is shown in Fig. 3. Details of the diamond Raman laser and the method used to generate an unpolarized input beam are outlined below in the Methods section.

**Figure 4:**
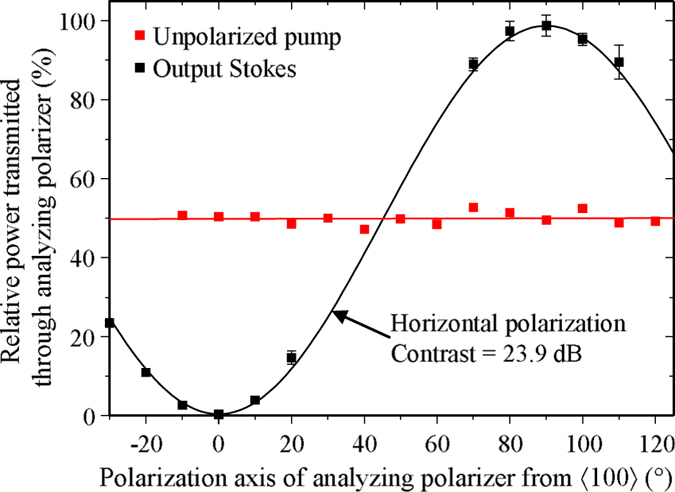
Percentage of input pump and output Stokes power transmitted through an analyzer as a function of angle. Error bars indicate the standard deviation of repeated measurements and solid lines indicate fits used to calculate the polarization contrast of the pump and converted Stokes signals.

For an unpolarized input beam, the output polarization of the Raman laser was purely horizontal. The polarization contrast ratio of the Stokes output beam (determined from the squared cosine fit of the transmitted power through an analysing polarizer as a function of analyser angle) was greater than 23.9 dB, as shown in Fig. 4. The contrast was limited by the precision of the measurement and is indicative of SRS of a single polarization mode.

**Figure 5:**
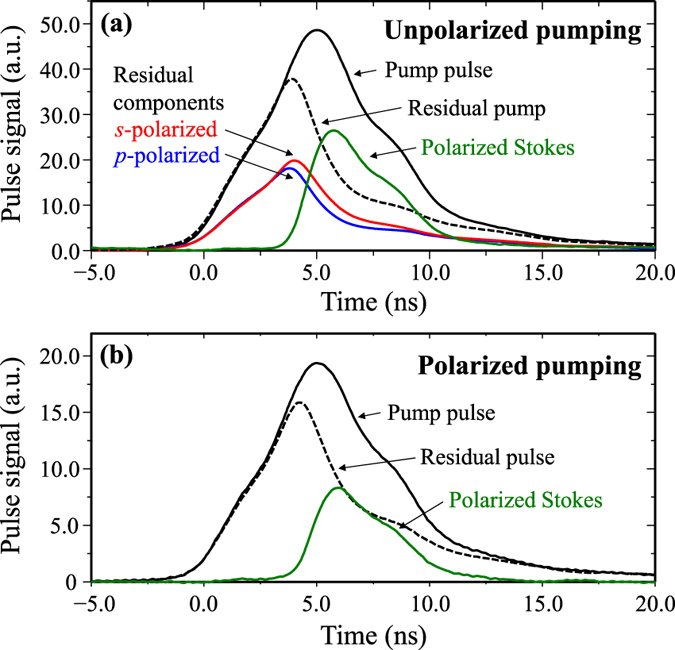
Pump, residual pump and Stokes pulses for (**a**) unpolarized and (**b**) polarized pumping.

In order to confirm non-trivial polarization conversion, the power transfer from the two input polarization components were measured using sampling wedges placed in the interferometer arms at locations 1 and 2 in Fig. 3. Oscilloscope traces were recorded for the incident and residual pump components and Stokes output pulses (Fig. 5). The *s*- and *p*-polarized components of the residual pump beam, shown in Fig. 5(a), are approximately equally depleted (52.9% of the total residual pump is *s*-polarized and 47.1% is *p*-polarized) demonstrating coupling of both polarization components from the unpolarized pump beam, in agreement with the near unity value for *g*_⊥_/*g*_||_ shown in Fig. 2.

**Figure 6:**
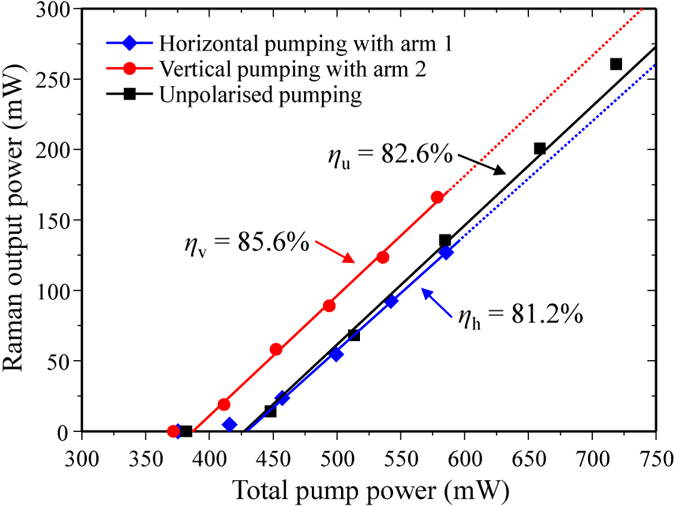
Stokes output power of the diamond Raman laser for horizontally- and vertically-polarized and unpolarized pumping conditions. Horizontally-polarized pump was generated with arm 2 of the interferometer blocked, and vertically-polarized pump with arm 1 blocked.

**Figure 7:**
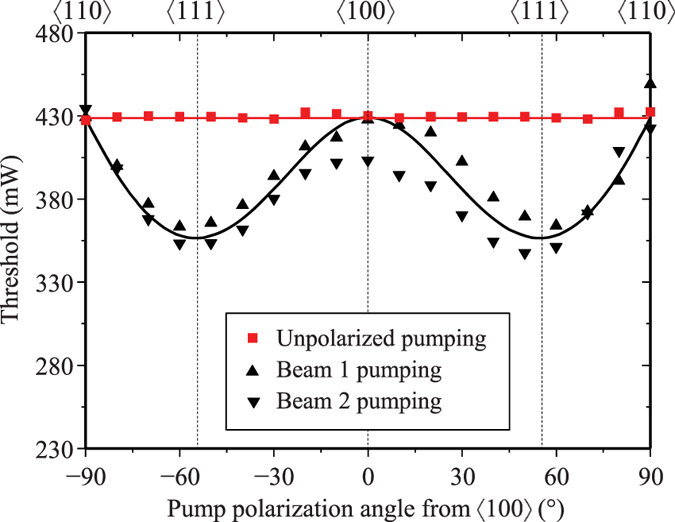
Stokes threshold for pump polarization principle angles from the [100] axis in diamond. Uncertainty in each threshold measurement was 10%. Black and red lines show trends determined from the inverse of (3) for polarized and unpolarized pumping.

The strong coupling of both input polarizations enables a high slope efficiency to be achieved with the unpolarized input. The slope efficiency shown in Fig. 6 approached the quantum defect limit of the Raman process for first Stokes generation of 85.8%, illustrating efficient Raman conversion regardless of pump polarization. This is almost identical to the slope efficiencies obtained for linear polarized pumping (see also Fig. 6), and enables an overall conversion efficiency of 36% to be obtained. In the present case, the overall conversion efficiency is limited by the quantum defect of the Raman process and the modest range of operation above laser threshold. Nevertheless, the high slope efficiency reveals the high degree of cooperative coupling of the two input polarizations. In order to further demonstrate the cooperative action of the input polarization components, the laser threshold was measured as a function of the final pump half-wave plate angle (HWP4 in Fig. 3). The threshold was determined by extrapolating power values at two different pump levels well above threshold and tracing linearly back to the threshold value. As shown in Fig. 7, equal thresholds were obtained for linear pump polarizations aligned parallel to the [100] and [110] axes and for the unpolarized pump, as predicted in Fig. 1(d). For linearly polarized pumping, the threshold varies with polarization in reasonable accordance with that previously reported^22^, and also the Mueller calculus described earlier. Some difference is noted between the threshold behaviour for beams 1 and 2 seen in Fig. 7. This is attributed to slightly different alignments of the two input polarizations with the Stokes resonator axis. The lowest threshold was achieved for pump polarizations aligned to the <111> as expected for the enhanced gain conditions^22^. As shown in Fig. 7, the threshold remains constant for unpolarized pumping, at 430 mW. Along with the measured slppe efficiency of 83% (Fig. 6), the characteristic laser performance is almost identical to that observed for horizontally polarized pumping (threshold of 430 mW and slope 81%). Since Raman gain is inversely proportional to threshold, Fig. 7 highlights approximately equal gain is obtained for vertically, horizontally and unpolarized inputs.

## Discussion

This work has shown that cubic Raman crystals with *E* and *F* Raman modes provides a means for efficient non-trivial polarization conversion. On the other hand, cubic crystals with *A* Raman modes offer little prospect of coupling the intensity of orthogonal polarizations into a single linearly polarized beam. This is in agreement with measurements in barium nitrate, which has a strong Raman mode having *A*-class Raman symmetry^43^ and has only demonstrated polarized Stokes beams from linear and circularly polarized pumps^44^,^45^.

The polarization conversion provided by cubic Raman materials with more than one degenerate Raman mode is a significant advance for enabling efficient frequency conversion of a greater diversity of pump sources. High power fiber lasers are often unpolarized unless more complicated fiber designs are used and many solid-state lasers at increased power levels become depolarized due to thermally-induced birefringence. The recently demonstrated capability of diamond Raman lasers to generate high average power without saturation (380 W^26^) thus makes them highly suitable for converting such lasers. As a result of this applicability to unpolarized pumps, in combination with the Raman benefit of avoiding the thermal dephasing problem of *χ*^(2)^ processes, diamond conversion may be a critically important future technology for frequency downconversion at high average powers. The linear polarized output state is ideal for many applications as well as for further downstream nonlinear frequency conversion and other polarization dependent operations such as polarization combination.

Raman crystals other than diamond that possess *F*-type symmetry, such as single-crystal silicon and germanium, will show analogous polarization properties when using unpolarized pumps. Silicon Raman devices have received significant attention in the past decade due to their potentially high gain and infrared transmission, and compatibility with mature semiconductor manufacturing techniques and their resulting promise for compact (on-chip) devices^15^,^16^,^17^,^18^,^46^ although some progress has been hampered by large multiphoton effects^47^. Future development of bulk and waveguide silicon Raman lasers may also benefit from the polarization conversion concept outlined herein.

In conclusion, we have demonstrated polarization conversion of an unpolarized pump to a linearly polarized Stokes with a slope efficiency approaching the quantum defect of the Raman process and similar to the maximum obtained for linearly polarized pumping. Based on Mueller calculus, we show that the polarization-dependent spontaneous Raman scattering defines a preferred polarized seed orientation and that all input polarization components are efficiently transferred to a pure linear output polarization mode. Furthermore, within the cubic crystal class, only crystals with degenerate *E* and *F* modes possess the required symmetry to provide such cooperative coupling. We expect that the results will greatly diversify the application of Raman lasers to unpolarized or depolarized pumps often characteristic of high-power lasers.

## Methods

The pump laser is a 1064-nm Q-switched laser (ESKPLA; model NL-220) with 6 ns pulses (FWHM) at 1-kHz pulse repetition rate. The beam quality was *M*^2^ ≈ 1.1 and was linearly polarized. A half-waveplate (HWP) and an optical isolator were used directly after the pump laser to manipulate the pump power in addition to limiting the amount of feedback into the pump laser due to strong back reflections from double-pass pumping of the diamond Raman laser.

After isolation, additional waveplates and polarizing cubes (PBS) split the pump beam into two paths with equal intensities and then recombines them to form an unbalanced interferometer. One arm of the interferometer is delayed by approximately 7.5 cm, as shown in Fig. 3, which is many times longer than the coherence length of the pump laser (<0.5 cm), and thus ensures, when recombined, that the pump beam is unpolarized. The polarization state of the recombined beam was investigated further using an analysing polarizer which transmitted constant power across all waveplate rotation angles as shown in Fig. 4, confirming an unpolarized state of the recombined pump beam. Blocking either arm (at locations 1 and 2 in Fig. 3) produced a linearly polarized beam of half intensity with a polarization contrast of greater than 20 dB.

The external cavity Raman laser was formed using a 5-cm radius-of-curvature mirror and a planar mirror (M1 and M2 respectively) each with dielectric-coatings optimized primarily for the first Stokes of diamond at 1240 nm and double pass pumping, similar to the resonator described in ref. 35. The output coupling at 1240 nm was 60% and transmission at 1485 nm on both mirrors was greater than 80% to delay the onset of cascading to higher Stokes orders. A lens (L) with a focal length of 300 mm was used to focus the pump beam into the diamond with a beam waist diameter of 150 *μ*m. The confocal parameter of the pump beam was longer than the approximately 2 cm-long resonator. The diamond was an 8 mm-long single-crystal (ultra-low-birefringence, Element Six, UK). The propagation direction was along a <110> axis, which maximizes both Raman gain and DOP for unpolarized pumping (Fig. 1), as well as a coupling ratio of unity for orthogonal input polarizations, as shown in Fig. 2. The Raman laser was aligned so that pumping with either interferometer arm independently produced an equal threshold to within ~10% using the same polarization, confirming good overlap of combined pump beams. Furthermore, the range of pump powers used (as illustrated in Fig. 6) were such that only first Stokes emission at 1240 nm was observed.

## Additional Information

**How to cite this article**: McKay, A. *et al*. Polarization conversion in cubic Raman crystals. *Sci. Rep.* **7**, 41702; doi: 10.1038/srep41702 (2017).

**Publisher's note:** Springer Nature remains neutral with regard to jurisdictional claims in published maps and institutional affiliations.
